# Observations on the Inactivation Efficacy of a MALDI-TOF MS Chemical Extraction Method on *Bacillus anthracis* Vegetative Cells and Spores

**DOI:** 10.1371/journal.pone.0143870

**Published:** 2015-12-03

**Authors:** Simon A. Weller, Margaret G. M. Stokes, Roman A. Lukaszewski

**Affiliations:** CBR Division, Defence Science and Technology Laboratory, Ministry of Defence, Porton Down, Salisbury, United Kingdom; University of Connecticut, UNITED STATES

## Abstract

A chemical (ethanol; formic acid; acetonitrile) protein extraction method for the preparation of bacterial samples for matrix assisted laser desorption ionisation time-of-flight mass spectrometry (MALDI-TOF MS) identification was evaluated for its ability to inactivate bacterial species. Initial viability tests (with and without double filtration of the extract through 0.2 μM filters), indicated that the method could inactivate *Escherichia coli* MRE 162 and *Klebsiella pneumoniae* ATCC 35657, with or without filtration, but that filtration was required to exclude viable, avirulent, *Bacillus anthracis* UM23CL2 from extracts. Multiple, high stringency, viability experiments were then carried out on entire filtered extracts prepared from virulent *B*. *anthracis* Vollum vegetative cells and spores ranging in concentration from 10^6^-10^8^cfu per extract. *B*. *anthracis* was recovered in 3/18 vegetative cell extracts and 10/18 spore extracts. From vegetative cell extracts *B*. *anthracis* was only recovered from extracts that had undergone prolonged Luria (L)-broth (7 day) and L-agar plate (a further 7 days) incubations. We hypothesise that the recovery of *B*. *anthracis* in vegetative cell extracts is due to the escape of individual sub-lethally injured cells. We discuss our results in view of working practises in clinical laboratories and in the context of recent inadvertent releases of viable *B*. *anthracis*.

## Introduction

Matrix-assisted laser desorption / ionisation time-of-flight mass spectrometry (MALDI-TOF MS) is used in clinical microbiology for the identification of bacterial or fungal strains isolated from positive blood cultures [1]. The mass spectra produced from isolates are reproducible, quickly generated, and libraries of spectra have been created from the analysis of known strains allowing the rapid identification of unknown isolates.

Sample preparation can simply involve the transfer of isolated culture (e.g. with a toothpick) to the target plate and overlaying with HCCA matrix (α-Cyano-4-hydroxycinnamic acid)—a direct sample application. Chemical extraction methods can also be performed and have been shown to increase the number of successful identifications, especially for Gram-positive bacteria [2]. On a target plate 1 μL volumes of chemical extract are dried, overlain with 1 μL matrix, and tested.

Routine use of MALDI-TOF systems in hospital laboratories typically involves the use of a direct method to apply culture to target plates on the laboratory bench. MALDI-TOF systems are generally not operated within primary biological safety containment (i.e. a microbiological safety cabinet). The vast majority of organisms isolated from hospital blood cultures are classed in the UK as Advisory Committee on Dangerous Pathogens (ACDP) Hazard Group (HG) 2 or lower, and therefore pose a low risk to the operative. However, there is a danger that an operative may inadvertently apply an ACDP HG3 bacterial agent directly to a target plate, especially in a geographic region where ACDP HG3 organisms are endemic. This may incur an unacceptable risk of exposure to the operative.

MALDI-TOF chemical extraction methods have been reported to inactivate ACDP HG3 bacteria, though the addition of filtration sterilisation steps has been reported to be required to ensure all viable bacteria are removed from the extract [3, 4], especially samples which may contain bacterial spores. A recent inadvertent release of *B*. *anthracis* in a US laboratory was attributed to the production of an unfiltered MALDI-TOF chemical extract [5].

In this paper we report on the inactivation efficacy of a MALDI-TOF chemical extraction method (using ethanol, formic acid, acetonitrile, and filtration), on the viability of high concentrations of *Bacillus anthracis* vegetative cells and spores. This method was designed to be able to be used in a clinical laboratory, within a small microbiological safety cabinet or isolator, with the MALDI-TOF MS system being situated on the bench, outside of primary biocontainment. The method was required to allow generation of mass spectra which could be compared with databases supplied with the Bruker Biotyper MALDI-TOF MS system, and therefore allow routine identification of common clinical strains.

## Materials and Methods

### Chemical extraction method for MALDI-TOF MS analysis

The Formic Acid Extraction Method, as found in the Biotyper manufacturers (Bruker) user manual [6], was adapted to include a filtration step. In brief: 10 μL loops of culture were added to 1 mL of 70% ethanol. The suspension was mixed thoroughly, centrifuged (13 000 rpm; 2 min), and the supernatant discarded. The pellet was then allowed to air dry. The pellet was then re-suspended in 50 μL of 70% formic acid and mixed thoroughly. Fifty μL of 100% acetonitrile was then added, the suspension mixed thoroughly, and centrifuged (13 000 rpm; 2 min). The supernatant was then passed (7 000 rpm; 10 secs) through a 0.2 μm Micro-Spin, Regenerated Cellulose membrane, spin column (Chrom Tech Inc, MN). The resulting filtrate was then passed (7 000 rpm; 10 secs) through a fresh 0.2 μm Micro-Spin spin column and the filtrate retained for MALDI-TOF analysis.

Regenerated cellulose filter membrane spin columns were chosen for their low protein binding characteristics and reported resistance to solvents [7], plus their ability to allow filtration of low sample volumes (< 100 μL). All reagents (Sigma-Aldrich UK) were reagent grade or HPLC grade. The method was tested using overnight cultures of the avirulent (pXO1^-^; pXO2^-^) *B*. *anthracis* UM23CL2 strain [8]. With input amounts in the range of 10^7^ to 10^8^colony forming units (cfu) resulting protein extracts were shown to be able to be identified as *B*. *anthracis* when resulting mass spectra were compared against spectra in the Bruker Security Relevant [9] database (data not shown). *E*. *coli* MRE 162 and *S*. *aureus* ATCC 6538 strains, species commonly isolated from blood culture, were also successfully identified from extracts produced using the method (when spectra were compared with those in the Bruker Taxonomy database).

### Initial evaluation of inactivation efficacy of method (with and without filtration) against gram-negative and gram-positive bacteria

Under ACDP containment level (CL) 2 (BSL-2 in other countries) conditions *Escherichia coli* MRE 162, *Klebsiella pneumoniae* ATCC 35657, and *Bacillus anthracis* UM23CL2 were incubated (37°C) overnight on Luria (L) agar plates. In each species specific experiment 10 μL microbiological loops of culture were extracted using the above method (3 reps with filtration / 3 reps without filtration). Entire protein extracts (typically comprising final volumes of 80–90 μL) were pipetted into the wells of a sterile 6-well tissue culture plate (Corning Costar), and spread around the base of each well with a sterile cell scraper. Extracts were then allowed to dry (typically for 20–30 mins) in order to ensure the formic acid and acetonitrile evaporated and was not carried forward into culture. Initial work had shown that even low concentrations (0.05% each) of these chemicals could interfere with L-broth bacterial culture (data not shown). Each experiment (i.e. each species) also included a positive and negative control. Positive controls comprised cell suspensions prepared following the above method—until the filtration steps–but with substitution of all reagents with 1 × Dulbeccos Phosphate Buffered Saline (1 x DPBS). The final bacterial pellet (in 100 μL of 1 x DPBS) was re-suspended and added to a well, as before, and allowed to dry. A sterile swab was applied to the dried protein extract / positive control visible in the bottom of each well in a repetitive crossing pattern and then streaked onto L-agar plates. Negative controls comprised a swab applied to a naïve plate well. Plates were incubated (48 hours; 37°C).

Resulting colonies from the *B*. *anthracis* extracts were re-isolated onto fresh L-agar plates and incubated (24hr; 37°C). One μL of resulting culture was added to 1 mL sterile distilled water and heated (99°C; 15 mins). One μL aliquots of the resulting lysed cell suspensions were tested by PCR on the chromosomal target Ba chr-MGB -[10].

### High stringency evaluation of the inactivation efficacy of the method on *Bacillus anthracis* Vollum vegetative cells and spores

Under ACDP CL3 conditions the virulent (pXO1^+^; pXO2^+^) *B*. *anthracis* Vollum strain was cultivated overnight (37°C) in either L-broth or on L-agar plates. None of the cultures used were more than 24 hours old to help prevent widespread sporulation on agar plates. Cell pellets or plate cultures were re-suspended into 1 mL of sterile 1 × DPBS, and 100 μL aliquots of the resulting suspension were added to 900 μL of 70% Ethanol. MALDI extracts were then prepared using formic acid and acetonitrile, and double filtration, as described above. Suspensions were enumerated (to determine the number of bacterial cells in 100 μL aliquots), by the production of a 10-fold dilution series and plating of appropriate dilutions (100 μL aliquots; 3 reps per dilution) onto L-agar plates. These plates were incubated (min. 48 hours) and colonies counted. Extracts were also prepared from a pre-existing, enumerated (1.2 × 10^9^ cfu·mL^-1^), suspension of *Bacillus anthracis* Vollum spores.

In total eighteen MALDI-TOF extract replicates were produced from each cell type. Each experiment comprised 3 MALDI-TOF protein extracts (from an overnight plate culture or the pre-existing spore suspension), one positive control and one negative control. Different experiments were initiated on different days. Extracts / positive controls were applied to individual wells of sterile tissue culture plates, allowed to dry and then re-suspended by adding 100 μL aliquots of sterile 1 × DPBST (DPBS with 0.01% Tween) to each well. The aliquot was applied across the well by repeated pipetting (30 secs) to ensure coverage of the entire well base and re-suspension of as much protein extract / positive control as possible. All 1 × DPBST was then removed from each well and put into culture (see below). A negative control (addition of 100 μL of sterile 1 × DPBST to a naïve well) was also prepared and cultured in each experiment.

To provide a qualitative indication (QUAL experiments) to the inactivation efficacy of the method nine reps of each cell type protein extract (re-suspended into 1 × DPBST) were added to 900 μL of Luria broth and incubated (7 days; 37°C). Entire broths were then plated onto L-agar (250 μL × 4 plates) and incubated (a further 7 days; 37°C).

To provide a quantitative indication (QUANT experiments) on the inactivation efficacy of the method, nine reps of each cell type protein extract (re-suspended into 1 × DPBST) were directly plated onto L-agar plates and incubated (4–7 days; 37°C).

Resulting colonies from vegetative cell extracts were re-isolated onto fresh L-agar plates and incubated (24hr; 37°C). Where there were too many cells to count (TMTC) a 1 μL loop was streaked across the centre of the plate and used to inoculate a fresh L-agar plate. A 1 μL loop of each resulting culture was added to 1 mL sterile distilled water and heated (99°C; 15 mins). One μL aliquots of the resulting lysed cell suspensions were tested by PCR on the pXO1 target pXO1-MGB [10]. Resulting colonies on L-agar plates from spore extracts were either tested by the same method or assumed to be *B*. *anthracis* on the basis of typical colony morphology.

## Results

### Initial inactivation experiments (with and without filtration)

Results from the initial inactivation experiments are summarised in Table 1. No input strain was recovered from filtered or non-filtered extracts generated from *E*. *coli* MRE162 or *K*. *pneumoniae* ATCC 3657 strains. *B*. *anthracis* UM23CL2 was recovered from 2/3 non-filtered extracts, as confirmed by Ba chr-MGB PCR [10], but not from any filtered extracts.

**Table 1:** Summary of input strain recovery on L-agar plates streaked with dried MALDI protein extracts (generated from viable cultures) during initial inactivation experiments.

Input strain	Sample	Recovery of input strain on L-agar plates		
		Extract 1	Extract 2	Extract 3
*B*. *anthracis* UM23CL2	Filtered Extracts^1^	No	No	No
	Non-filtered Extracts	No	YES^2^	YES^2^
	Positive control	YES	n/a^3^	n/a
	Negative control	No	n/a	n/a
*E*. *coli* MRE 162	Filtered Extracts	No	No	No
	Non-filtered Extracts	No	No	No
	Positive control	YES	n/a	n/a
	Negative control	No	n/a	n/a
*K*. *pneumoniae* ATCC 35367	Filtered Extracts	No	No	No
	Non-filtered Extracts	No	No	No
	Positive control	YES	n/a	n/a
	Negative control	No	n/a	n/a

^1^ Extracts double filtered through 0.2 μM filter membranes.

^2^ Confirmed as *B*. *anthracis* Ba chr-MGB PCR (Parsons *et al*., 2013)

^3^ n/a = not applicable—only one positive and negative control per experiment.

### *B*. *anthracis* Vollum inactivation experiments

Results from vegetative cell experiments are summarised in Table 2. *B*. *anthracis* was recovered from 3/18 replicates and only from QUAL experiments where replicates had undergone a L-broth recovery incubation (7 days), and a further L-agar plate incubation (a further 7 days). In one replicate (QUAL MALDI 1: Extract 3), seven individual *B*. *anthracis* colonies were recovered from one plate replicate, rather than the multiple *B*. *anthracis* colonies recovered from all four plates in the other two positive replicates (QUAL MALDI 3: Extracts 2 and 3). Individual colonies and plate cultures from these experiments were confirmed as *B*. *anthracis* by pXO1 MGB-PCR.

**Table 2:** Summary of *B*. *anthracis* recovery, from dried MALDI protein extracts derived from vegetative cells, in QUAL and QUANT experiments.

Experiment and *B*. *anthracis* input amount	Extract or control	*B*. *anthracis* L-agar plate colony counts / coverage estimate^a^			
		Plate 1	Plate 2	Plate 3	Plate 4
QUAL MALDI 1^b^	Extract 1	0	0	0	0
1.4 × 10^7^cfu	Extract 2	0	0	0	0
	Extract 3	0	0	7^d^	0
	Positive	TMTC (>95%)	TMTC (>95%)	TMTC (>95%)	TMTC (>95%)
	Negative	0	0	0	0
QUAL MALDI 2^c^	Extract 1	0	0	0	0
2.0 × 10^8^ cfu	Extract 2	0	0	0	0
	Extract 3	0	0	0	0
	Positive	TMTC (>95%)	TMTC (>95%)	TMTC (>95%)	TMTC (>95%)
	Negative	0	0	0	0
QUAL MALDI 3^c^	Extract 1	0	0	0	0
2.0 × 10^8^ cfu	Extract 2	TMTC (>95%)^d^	TMTC (>95%)^d^	TMTC (>95%)^d^	TMTC (>95%)^d^
	Extract 3	TMTC (>95%)^d^	TMTC (>95%)^d^	TMTC (>95%)^d^	TMTC (>95%)^d^
	Positive	TMTC (>95%)	TMTC (>95%)	TMTC (>95%)	TMTC (>95%)
	Negative	0	0	0	0
QUANT MALDI 1^b^	Extract 1	0	n/a^e^	n/a	n/a
6.7 × 10^6^ cfu	Extract 2	0	n/a	n/a	n/a
	Extract 3	0	n/a	n/a	n/a
	Positive	TMTC (>90%)	n/a	n/a	n/a
	Negative	0	n/a	n/a	n/a
QUANT MALDI 2^c^	Extract 1	0	n/a	n/a	n/a
6.7 × 10^8^ cfu	Extract 2	0	n/a	n/a	n/a
	Extract 3	0	n/a	n/a	n/a
	Positive	TMTC (>95%)	n/a	n/a	n/a
	Negative	0	n/a	n/a	n/a
QUANT MALDI 3^c^	Extract 1	0	n/a	n/a	n/a
5.8 × 10^8^ cfu	Extract 2	0	n/a	n/a	n/a
	Extract 3	0	n/a	n/a	n/a
	Positive	TMTC (>95%)	n/a	n/a	n/a
	Negative	0	n/a	n/a	n/a

^**a**^ TMTC = too many to count; Coverage estimate (in parentheses): basic estimate of total coverage of plate by bacterial colonies or lawn.

^b^ Extracted culture prepared by overnight broth culture.

^c^ Extracted culture prepared by overnight plate culture.

^d^ Confirmed as *B*. *anthracis* by pXO1-MGB [10].

^e^ n/a = not applicable. Only one L-agar plate, per rep, was inoculated in QUANT experiments.

Results from spore experiments are summarised in Table 3. *B*. *anthracis* was recovered from 10/18 replicates, including quantification (QUANT) experiments where extracts were not incubated in L-broth. Plate cultures from QUAL MALDI S2 and QUAL MALDI S3 experiments were confirmed as *B*. *anthracis* by pXO1-MGB PCR. In QUANT experiments the total number of colonies from 4/9 extracts comprised 5, 3, 5, and 107 colonies respectively. These colonies were either confirmed to be *B*. *anthracis* by pXO1-MGB PCR (QUANT S1 experiment) or assumed to be *B*. *anthracis* due to typical colony morphology (QUANT S2 experiment).

**Table 3:** Summary of *B*. *anthracis* recovery, from dried MALDI protein extracts derived from spores, in QUAL and QUANT experiments.

Experiment and *B*. *anthracis* input amount	Extract or control	*B*. *anthracis* L-agar plate colony counts / coverage estimate^a^			
		Plate 1	Plate 2	Plate 3	Plate 4
QUAL MALDI S1	Extract 1	0	0	0	0
1.2 × 10^8^ cfu	Extract 2	0	0	0	0
	Extract 3	0	0	0	0
	Positive	TMTC (>95%)	TMTC (>95%)	TMTC (>95%)	TMTC (>95%)
	Negative	0	0	0	0
QUAL MALDI S2	Extract 1	TMTC (>90%)^b^	TMTC (>90%)^b^	TMTC (>90%)	TMTC (>90%)
1.2 × 10^8^ cfu	Extract 2	TMTC (>90%)^b^	TMTC (>90%)^b^	TMTC (>90%)	TMTC (>90%)
	Extract 3	TMTC (>90%)^b^	TMTC (>90%)^b^	TMTC (>90%)	TMTC (>90%)
	Positive	TMTC (>90%)	TMTC (>90%)	TMTC (>90%)	TMTC (>90%)
	Negative	0	0	0	0
QUAL MALDI S3	Extract 1	TMTC (>95%)^b^	TMTC (>95%)^b^	TMTC (>95%)	TMTC (>95%)
1.2 × 10^8^ cfu	Extract 2	TMTC (>95%)^b^	TMTC (>95%)^b^	TMTC (>95%)	TMTC (>95%)
	Extract 3	TMTC (>95%)^b^	TMTC (>95%)^b^	TMTC (>95%)	TMTC (>95%)
	Positive	TMTC (>95%)	TMTC (>95%)	TMTC (>95%)	TMTC (>95%)
	Negative	0	0	0	0
QUANT MALDI S1	Extract 1	0	n/a^d^	n/a	n/a
1.2 × 10^8^ cfu	Extract 2	5^b^	n/a	n/a	n/a
	Extract 3	3^b^	n/a	n/a	n/a
	Positive	TMTC (>95%)	n/a	n/a	n/a
	Negative	0	n/a	n/a	n/a
QUANT MALDI S2	Extract 1	5^c^	n/a	n/a	n/a
1.2 × 10^8^ cfu	Extract 2	107^c^	n/a	n/a	n/a
	Extract 3	0	n/a	n/a	n/a
	Positive	TMTC (>95%)	n/a	n/a	n/a
	Negative	0	n/a	n/a	n/a
QUANT MALDI S3	Extract 1	0	n/a	n/a	n/a
1.2 × 10^8^ cfu	Extract 2	0	n/a	n/a	n/a
	Extract 3	0	n/a	n/a	n/a
	Positive	TMTC (>95%)	n/a	n/a	n/a
	Negative	0	n/a	n/a	n/a

^**a**^ TMTC = too many to count; Coverage estimate (in parentheses): basic estimate of total coverage of plate by bacterial colonies or lawn.

^b^ Confirmed as *B*. *anthracis* by pXO1-MGB PCR [10].

^c^ Assumed to be *B*. *anthracis* due to typical colony morphology.

^d^ n/a = not applicable. Only one L-agar plate, per rep, was inoculated in QUANT experiments.

## Discussion

*Bacillus anthracis* Vollum was chosen for the high stringency inactivation experiments based on the results from the initial inactivation experiments. These indicated that viable *B*. *anthracis* was harder to inactivate / remove in extracts than two gram-negative bacterial species (*E*. *coli* and *K*. *pneumoniae*). In addition it was possible to test *B*. *anthracis* Vollum in its spore form, considered to be one of hardest organism types to inactivate [11], which allowed as much information on the inactivation efficacy of the method to be obtained as was possible. It is unlikely that neat preparations of *B*. *anthracis* spores would be tested in a clinical laboratory as fresh plate cultures (from positive blood cultures) are typically tested. These are not cultivated on defined sporulation (nutrient deficient) media.

Previous research has indicated the requirement for filtration of MALDI-TOF extracts to ensure inactivation or removal of *Bacillus anthracis* [4], especially extracts that could contain spores—a finding confirmed in a recent Centers for Disease Control and Prevention report [5]. The stringent *B*. *anthracis* Vollum inactivation experiments conducted in our study differed in methodology from previous studies in that the entire protein extract was tested for the presence of viable *B*. *anthracis* and the formic acid and acetonitrile matrix was evaporated off to prevent interference with culture techniques carried out in viability testing. During method development we determined that even 0.05% concentrations of acetonitrile and formic acid in L-broth could interfere with the growth of *B*. *anthracis* (data not shown). In addition, in half of the experiments, a broth culture recovery incubation was applied in order to facilitate the best chance of the resuscitation of cells stressed or damaged during the inactivation method, but which retain viability.

In total, from all vegetative cell experiments, viable *B*. *anthracis* were recovered from 3/18 experimental replicates (QUAL and QUANT experiments). Viable *B*. *anthracis* cells were only recovered from replicates which underwent L-broth recovery incubations (QUAL experiments). It is hypothesised that the recovery of *B*. *anthracis* cells in these experiments represent the survival of individual cells that were sub-lethally injured or induced into a viable but not-culturable state (VBNC), by the inactivation method but which were able to be resuscitated during two weeks of culture. This hypothesis is supported by the isolation of 7 individual *B*. *anthracis* cells from one L-agar plate in the QUAL MALDI 1 (Rep 3) experiment. This suggests that these cells were sub-lethally injured to the point where they could not replicate during 7 days of broth culture but which were subsequently able to grow on L-agar plates.

In quantitative (QUANT) vegetative cell experiments, where no broth culture incubation was employed, no *B*. *anthracis* colonies were recovered. A previous study [12] has shown that disinfectant treated *Bacillus atropheaus* spores can be more readily identified during broth culture incubation than direct plate culture. Our study indicates that this may also be true of vegetative *B*. *anthracis* cells. If this is true, and the recovery of *B*. *anthracis* in the QUAL MALDI 1 (input 10^7^ cfu) and QUAL MALDI 3 (input 10^8^ cfu) experiments does represent the survival of sub-lethally injured or VBNC individual cells, then a six to seven log reduction in viable cell counts was observed. It should be noted that in 10 experimental replicates with an input of 10^8^ cfu (vegetative cells), no viable *B*. *anthracis* were recovered.

It is possible that recovery of *B*. *anthracis* from vegetative cell MALDI extracts might be a function of sporulation on L-agar plates prior to extraction, even with fresh cultures being processed. As might be expected, *B*. *anthracis* spores were shown to be more difficult to inactivate by the chemical extraction method with viable *B*. *anthracis* being recovered from 10/18 spore extract replicates. In quantification (QUANT) spore experiments, where inactivation was not demonstrated, the highest colony count from an extract was 107, suggesting that in this instance a six log reduction in viable spores was achieved. However, if individual spores are able to survive an extraction process, whatever the input amount, this may account for the recovery of *B*. *anthracis* extracts in the vegetative cell experiments.

It is striking that from extracts generated from both cell types a range of positive and negative *B*. *anthracis* recovery responses were generated. The multiple replicates tested in our study help elucidate this phenomena, as it has in other pathogen inactivation studies [13], but we have not determined what survival mechanism(s) are being employed by *B*. *anthracis*. We use terms such as ‘sub-lethally injured’ and ‘VBNC’ only as labels to hypothesise how *B*. *anthracis* might be surviving the extraction process without determining which, if either, of these labels might apply. Various stress responses are known to be employed by bacteria entering a VBNC state, including modifications to cell walls and membranes [14]. Significantly, bacterial cells are also known to shrink during a stress response [14]. Although filtration was indicated to be an important component of *B*. *anthracis* inactivation in this study (as it has in others) it appears that spores and vegetative cells were able to pass through two 0.2 μM filters. A gram-positive rod, *B*. *anthracis* vegetative cells range in size between 1–1.2 μM in diameter and 3–5 μM in length. *B*. *anthracis* spores have been measured with mean diameters 0.81–0.86 μM and mean lengths of 1.26–1.67 μM [15]. It is possible that the action of ethanol, formic acid, and acetonitrile could be shrinking the bacterial cell / spore allowing passage through filters, though other factors such as chemical action on the filter membrane or inconsistencies in the pore size could also play a part, as in fact could a combination of all these factors.

In our study the choice of a 0.2 μM filter size was mandated by an aspiration to use spin columns (for ease of use and the ability to filter low volumes), and the membrane material was chosen for its low retention of protein and for its reported resistance to solvents [7]. We could not source a 0.1 μM spin column filter and had we been able to do so the action of the solvent / acid test matrix on regenerated cellulose membranes is still unclear. With filtration identified as an important part of the sterilisation of MALDI extracts [3, 4, 5] a greater understanding of these factors (i.e. bacterial shrinkage; chemical effect on filter membranes; even operational factors associated with spin column architecture) could aid in the development of improved and robust inactivation protocols. As a pointer for future research we did not test filtrates from *B*. *anthracis* suspensions in water. This could help determine if there is a chemical action on filter membranes.

A further factor in the inactivation efficacy of the method is the volume of formic acid / acetonitrile required to produce a protein extract that is able to allow confident agent identification by MALDI-TOF MS. The manufacturer provides guidelines [6] that different amounts of these reagents can be used for different amounts of bacterial culture (i.e. 20–40 μL each for 1 μL culture loops; 40–80 μL each for 10 μL culture loops). In our study 50 μL volumes of formic acid were used as we had determined that amounts of *B*. *anthracis* of 10^7^ to 10^8^ cfu were required for a reliable identification by MALDI-TOF MS, and that these amounts were collected by these loops (data not shown). In terms of inactivation studies carried out under ACDP CL3 conditions it is expensive to test multiple different volumes against multiple different agent amounts. This is a potential difficulty in developing and validating a one-size-fits-all extraction method, allowing identification of common clinical strains and ACDP HG3 pathogens, but still inactivating all bacteria species and culture amounts required to be tested.

In this study 6 to 8 log reductions in viable bacterial counts were demonstrated when using the devised chemical extraction method. However, the highest quantified number of cells recovered from a 80–90 μL protein extract suspenion was 107 cells (QUANT MALDI S2: Extract 2), and this was from a neat spore suspension which is unlikely to be tested in a clinical laboratory. We have hypothesised that the recovery of *B*. *anthracis* from vegetative cell extracts represent the survival of sub-lethally injured or VBNC individual cells. With the requirement to only test a 1 μL volume of an extract (which is dried onto target plates and overlain with a further 1 μL HCCA matrix aliquot–with the HCCA being reconstituted in an acetonitrile / trifluoroacetic acid (TFA) solution), it is possible that methods using the findings of this project could be developed in clinical laboratories to provide only a very low residual risk to operatives. The risk is further lowered by the fact that the vast majority of clinical blood cultures do not contain ACDP HG3 pathogens. With MALDI-TOF being postulated as a driver for total laboratory automation [16] then an automated chemical extraction method could also further reduce the risk to the operative. If so then some of the findings in this study should be considered.

In summary, viability tests analysing the entire extract, with multiple replicates and extended broth and plate culture steps, has provided valuable information on the inactivation efficacy of the approach. In a separate study similar principles (i.e. testing of the entire extract; extended culture steps) were used to develop inactivation methods for Ebola Virus [13]. A recent report on the inadvertent shipment of *B*. *anthracis* spores by the US Department of Defense [17] has identified differences in viability testing methodology between institutes; e.g. some testing 10% of an extract, some omitting a broth culture stage. In previous reports [4, 5] on the bacterial inactivation efficiency of MALDI-TOF chemical extraction methods similar differences in methodology are also apparent, or have not been clearly defined. The findings of our study could therefore help inform the development of common Standard Operating Procedures for viability testing from many inactivated sample types–a recommendation in the US Department of Defense report—in addition to improving bacterial inactivation within MALDI-TOF protocols.
